# Comparing peripheral limb and forehead vital sign monitoring in newborn infants at birth

**DOI:** 10.1038/s41390-024-03651-0

**Published:** 2024-10-17

**Authors:** Suvvi K. Narayana Swamy, Simon J. Stockwell, Chong Liu, Caroline Henry, Lara Shipley, Carole Ward, Siavash Mirahmadi, Ricardo Correia, Stephen P. Morgan, John A. Crowe, Don Sharkey, Barrie R. Hayes-Gill

**Affiliations:** 1 Optics and Photonics Research Group and Centre for Healthcare Technologies, University of Nottingham, University Park, Nottingham, UK; 2 Centre for Perinatal Research, School of Medicine, University of Nottingham, Nottingham, UK

## Abstract

**Background:**

To study the feasibility of measuring heart rate (HR) and oxygen saturation (SpO_2_) on the forehead, during newborn transition at birth, and to compare these measurements with those obtained from the wrist.

**Methods:**

Vital signs were measured and compared between forehead-mounted reflectance (remittance) photoplethysmography sensor (fhPPG) and a wrist-mounted pulse oximeter sensor (wrPO), from 20 enrolled term newborns born via elective caesarean section, during the first 10 min of life.

**Results:**

From the datasets available (*n* = 13), the median (IQR) sensor placement times for fhPPG, ECG and wrPO were 129 (70) s, 143 (68) s, and 159 (76) s, respectively, with data recorded for up to 10 min after birth. The success rate (percentage of total possible HR values reported once sited) of fhPPG (median = 100%) was higher compared to wrPO (median = 69%) during the first 6 min of life (*P* < 0.005). Both devices exhibited good HR agreement with ECG, achieving >95% agreement by 3 (fhPPG) and 4 (wrPO) min. SpO_2_ for fhPPG correlated with wrPO (*r* = 0.88), but there were significant differences in SpO_2_ between the two devices between 3 and 8 min (*P* < 0.005), with less variance observed with fhPPG SpO_2_.

**Conclusion:**

In the period of newborn transition at birth in healthy term infants, forehead measurement of vital signs was feasible and exhibited greater HR accuracy and higher estimated SpO_2_ values compared to wrist-sited pulse oximetry. Further investigation of forehead monitoring based on the potential benefits over peripheral monitoring is warranted.

**Impact:**

- This study demonstrates the feasibility of continuously monitoring heart rate and oxygen saturation from an infant’s forehead in the delivery room immediately after birth.
- Significantly higher SpO_2_ measurements were observed from the forehead than the wrist during the transition from foetal to newborn life.
- Continuous monitoring of vital signs from the forehead could become a valuable tool to improve the delivery of optimal care provided for newborns at birth.

## Introduction

Approximately 10% of newborns require some form of stabilisation during the process of transition from prenatal foetal to postnatal newborn life.^1^ At birth, once the umbilical cord is cut there are rapid changes in the newborn’s cardiovascular and respiratory system as they transition to independent life.^2^ In high-risk infants, it is recommended to monitor a newborn’s vital signals such as heart rate (HR) and oxygen saturation (SpO_2_) continuously during this transition.^3^ The best indicator of the need and efficacy for resuscitation is the newborn HR.^4^ After effective breathing or ventilation is achieved it is recommended to monitor preductal oxygen saturations, in high-risk newborns.^5^ At birth, SpO_2_ has a marked variance that gradually rises typically from as low as 30–40%^6,7^ and in most cases, infants take upwards of 10 min to reach a stable period of SpO_2_ (>90%).^8–10^ A low or high SpO_2_ outside of target ranges is associated with adverse outcomes including death or significant brain injury.^9,11^ Therefore, it is essential to continuously monitor these vital signals during such a critical time as birth in the delivery room to provide optimal care for high-risk newborns.

Pulse oximeters (POs) are well-established, non-invasive optical devices for monitoring newborn vital signs, typically measured at peripheral sites on limbs. There are two main modes of PO usage: transmission and reflection.^12^ Transmission mode POs function by emitting light through peripheral locations such as the fingers, wrist, toes, earlobes, or the foot with a detector on the opposite side to measure the light transmitted. Reflection mode POs can be used on most parts of the body, with the detector and light source placed adjacent to each other. Peripheral monitoring using transmission mode PO is standard practice, yet it often faces challenges in the first minutes of life due to motion artefact^13^ and poor tissue perfusion.^14^ These issues can hinder the PO’s ability to accurately monitor vital signals, potentially delaying critical appropriate resuscitation.

Previous studies involving adults^15–17^ and newborns^18^ have shown that the forehead may offer a more suitable site for measuring both HR and SpO_2_ than at peripheral sites such as the finger or wrist. The central forehead region has many benefits, providing good quality and reliable pulsatile signals;^16^ it is less affected by temperature or vasoconstriction and rapidly detects deoxygenation;^16^ provides less interference with resuscitation procedures in a delivery room;^17^ and physiologically the blood supply is likely to be more reliable than peripheral blood supply during low perfusion states and transition.^13,14,19^ Thus, if newborn forehead HR and SpO_2_ measurements are more reliable it would allow the healthcare team to better understand the respiratory and cardiovascular status of the infant. Oxygenation at the forehead is also preductal, which plays a very important role in newborns for the detection of hypoxia and critical heart disease.^20^

Previously, we reported a preliminary study on the newborn’s forehead using a green reflectance (remittance) photoplethysmography (PPG) sensor (fhPPG) for effective HR measurement in the Neonatal Intensive Care Unit (NICU).^17^ For instance, in newborns >=32 weeks gestation, the HR reliability and accuracy indicated by the positive percent agreement (PPA), was found to be 97.7% (at ±10 bpm agreement level) and the bias (LoA) was 0.0 (8.39) bpm, respectively. This fhPPG sensor, now embedded in a flexible T-shaped cap, has demonstrated its HR accuracy and reliability across a range of healthy neonates in the NICU (PPA = 99.6% and bias (LoA) = −0.6 (5.5) bpm) and in the delivery room (PPA = 98.6% and bias (LoA) = −0.5 (8.2) bpm).,^21^ as well as in critically unwell infants in the neonatal intensive care (PPA = 98.87% and bias (LoA) = 0.22 (8.44) bpm).^22^ In the former trial, the sensor contained two additional optical wavelengths – namely red and infra-red (IR) making it possible to assess and evaluate the ability of this cap to measure forehead SpO_2_. We aimed to study the feasibility and compare HR and SpO_2_ measurements using a forehead-based fhPPG sensor against a wrist-based pulse oximetry sensor (wrPO) during the first 10 min of life after birth.

## Materials and methods

### Study population

This prospective observational study was conducted in two tertiary maternity hospitals at Nottingham University Hospitals NHS Trust, UK, following ethical approval (NHS Health Research Authority Yorkshire & The Humber, Sheffield Research Ethics Committee 15/YH/0522). Prior to enrolment in the study, written informed parental consent was obtained, and assessments were conducted on 20 infants born by elective caesarean section (ECS). The inclusion criteria were infants ≥37-week gestation with no anticipated need for support at birth and delivered by planned ECS. The exclusion criteria were subjects who were deemed clinically unstable by clinicians, and receiving any type of stabilisation or palliative care. Standard care pathways meant the newborn was first shown to the parents and then observed on the neonatal resuscitaire to keep warm until the mother’s operation was complete.

### Study design

Immediately after birth, a suitably sized fhPPG sensor cap was placed on the infant’s head with data transmitted via Bluetooth to a USB hub. The fhPPG sensor consists of a biomonitoring sensor chip (BIOFY SFH7050), which houses the LEDs and photodiodes in reflection mode configuration. The sensor chip includes 3 wavelength channels – green (λ = 525 nm), red (λ = 660 nm), and infra-red (λ = 950 nm) to illuminate light onto the tissue. It also features a single photodetector with a spectral sensitivity ranging between 400 nm and 1100 nm to capture the light reflected back from the tissue. All 3 wavelength channels are sampled at 100 Hz with a 30 Hz anti-aliasing filter (switched capacitor filter with roll-off 20 dB/decade). A photograph of this fhPPG sensor on a mannequin, illustrating its placement on the newborn is shown in Supplementary Fig. 1. Three neonatal ECG electrodes were placed on the newborn’s chest (PD50-F4C, SKINTACT, Leonhard Lang GmbH) and a transmission mode PO sensor (LNCS Neo, Masimo) placed on the right wrist (wrPO). The ECG and wrPO sensors were pre-connected to a CARESCAPE Monitor B450 (General Electric Healthcare) incorporating both an ECG module and a Masimo signal extraction technology (SET) module for measuring SpO_2_. The raw data were recorded for up to 10 min until the attending team were happy for the infant to be held by the parents. To help with training and contextual information (e.g., sensor being moved), a webcam (Logitech C615) was placed above the resuscitaire. The three data streams (i.e., B450 monitor, fhPPG and webcam) are combined via a USB hub to a Windows 10 laptop (LenovoY50 2037) where it is displayed in real time (LabVIEW 2014) and stored. A detailed illustration of the entire set-up that shows the arrangement of all the equipment in place and the baby on the resuscitaire, is included in Supplementary Fig. 2.

The B450 monitor acquires HR data from both the ECG and wrPO based on the previous 12 s window, updated every 5 s. The raw fhPPG data were processed offline to generate the HR using the green channel. HR was calculated using the formula shown in Eq. 1, where the time period is estimated based on the interval between detected peaks. SpO_2_ was determined by analysing the red and IR channels, as this is a standard practice to estimate the R-ratio using Eq. 2. Subsequently, the R-value was converted into SpO_2_ using a commonly employed linear equation, as shown by Eq. 3.^23^

1 $${HR}=\frac{60}{{median}({{{\rm{time\; period}}}})}{bpm}$$

2 $$R=\frac{{{PI}}_{{red}}}{{{PI}}_{{IR}}}$$

3 $${{SpO}}_{2}=110-25\times R$$

The R-ratio represents the ratio of the perfusion indices at the red and IR channels and is inversely correlated to SpO_2_. To ensure data rate consistency between wrPO and fhPPG, HR and SpO_2_ values were generated from the fhPPG at the same frequency every 5 s based on the previous 12 s window length.

### Data analysis

Data analysis was performed using Matlab software (Matlab R2021a, The MathWorks Inc.). Continuous data were expressed as mean (standard deviation) or median (interquartile range, IQR), based on their distribution. *Success Rate* was calculated as the percentage of total data points available compared to the total possible number of data points during the sensor’s ON time. *HR accuracy* performance of fhPPG and wrPO against the reference ECG HR utilised two methods: *Positive Percent Agreement (PPA)* and *Root Mean Squared Error (RMSE)*. PPA was computed as a percentage of the time the test device (either fhPPG or wrPO) generated a valid HR within ±10% of the paired ECG HR.^21,24^ RMSE was calculated for paired HR values (fhPPG vs ECG and wrPO vs ECG). The significance of the association between the availability of HR data points within fhPPG and wrPO at each minute was examined using Fisher’s exact tests. The Wilcoxon rank sum test was utilised to evaluate statistical significance within the cohort-level distributions for both HR and SpO_2_ separately, for each minute and between devices, with a significance level set at *P* < 0.05. To account for multiple tests, adjustments were made to the significance level using the Bonferroni correction method (adjusted *P* < 0.005). To evaluate the strength and direction of the relationship between fhPPG R against wrPO SpO_2_, a linear regression was performed on cohort-level paired data. The RMSE and the Spearman correlation coefficient (r) were used to indicate the accuracy of the first-order polynomial model and correlation strength between the two continuous variables, respectively. A Bland–Altman (BA) analysis was conducted to compare the SpO_2_ values generated by the two devices. The difference between the paired SpO_2_ values (fhPPG − wrPO) was plotted against their average ([fhPPG + wrPO]/2). The bias was calculated by taking the average of these paired differences, and the limits of agreement (LoA) were determined by adding and subtracting two times the standard deviation from the bias. For all SpO_2_ statistical analyses, data were included only if the corresponding HR was within ±10% of the paired ECG HR; otherwise, it was discarded.

It should be noted that all three sensors—fhPPG, ECG and wrPO—were placed sequentially in this exact order. Initially, the data from the sensors were synchronised by aligning the calculated HR values. Then, comparisons and analyses for HR and SpO_2_ between devices were conducted during the period when all the devices were in place, relative to the time of birth.

## Results

A total of 20 newborns born via ECS were assessed. Data from seven newborns were excluded for the following reasons: infants required the cap to be removed for resuscitation or requiring interventions by the attending team (*n* = 5), loss of wireless signal (*n* = 1) and incorrect placement of devices (*n* = 1). Data were collected for up to 10 min on the remaining infants or until the clinical team decided the study procedure should end. The demographic characteristics of the infants included in the study (*n* = 13) are as follows: median (IQR) gestational age was 39^+2^ weeks (38^+2^−39^+3^ weeks), and birthweight was 3310 grams (2870−3625 grams). Five infants were male, eleven were Caucasian, one Mixed White/Afro Caribbean and one was Asian ethnicity. No infants received any form of breathing support.

### Heart rate measurement

**Fig. 1:**
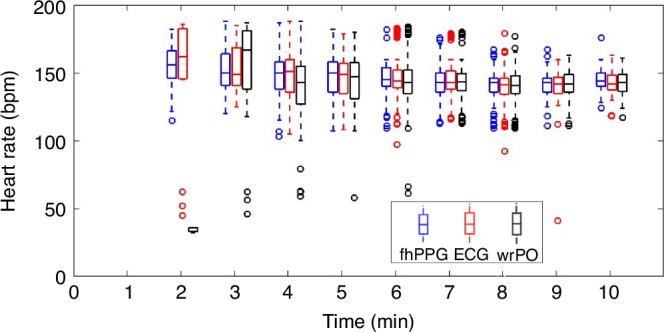
Box and whisker plots of cohort-level HR recorded over 10 min post-birth as measured by fhPPG, ECG and wrPO sensors (devices are offset for clarity). The line within the box represents the median value or the 50th percentile. The upper and lower edges of the box represent the lower quartile (25th percentile) and upper quartile (75th percentile), respectively. The IQR is defined as the difference between upper and lower quartiles. The T-shaped lines, known as whiskers, extend to the largest and smallest data points within ±1.5 × IQR from the upper and lower quartiles, respectively. Any data points that lie outside the range defined by the whiskers are considered outliers.

The median (IQR) placement time for fhPPG, ECG and wrPO sensors (placed sequentially in this order) in the overall cohort was 129 (70) s, 143(68) s and 159 (76) s after birth, respectively (Supplementary Table 1). Placement time is defined as the moment when the sensor was sited on the newborn and turned on. These timings were verified by cross-referencing the webcam footage with the time of birth (to the second ideally) recorded on the case report form. Box and whisker plots in Fig. 1 compare cohort-level heart rate (aligned HR data from fhPPG, ECG and wrPO measurements). Data were first obtained starting at 2-min after birth with a median (IQR) HR of 166 (36) bpm for ECG, 155 (28) bpm for fhPPG, and 36 (3) bpm for wrPO. The HR at 2 min was significantly lower for wrPO compared to ECG (*P* = 0.007), although there were only three wrPO measures (Supplementary Table 2).

**Fig. 2:**
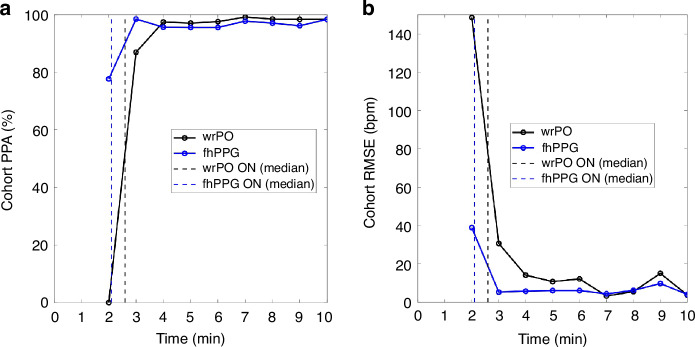
Comparison of HR accuracy for fhPPG and wrPO against reference ECG HR in the first 10 minutes post-birth. **a** Minute-by-minute cohort PPA (%). **b** Minute-by-minute cohort RMSE (bpm). Note that wrPO at 2 min has only 3 data points as it was placed after the ECG and the data are shown for completeness.

Success rates for HR for fhPPG, as shown in Table 1, was 100% throughout the recorded time period. Conversely, the success rate for wrPO at minute 2 was 11%, gradually increasing and reaching 100% by 7 min of life. Statistically significant differences in the proportion of available data points between fhPPG and wrPO were observed up to the first 6 min of life (*p* < 0.005). To quantify device accuracy, PPA and RMSE were calculated based on comparing to ECG-based HR measurements. The cohort-level PPA reached >95% for fhPPG at 3 min and wrPO at 4 min of age (Fig. 2a). The RMSE for both fhPPG and wrPO demonstrated improvement between minutes 2 and 10, reducing from 39 bpm to 4 bpm for fhPPG and from 149 bpm to 4 bpm for wrPO (Fig. 2b).

**Table 1:** Comparison of HR success rate between fhPPG and wrPO sensors within the first 10 min post-birth.

Time (min)	fhPPG % (data points available/total possible data points)	wrPO % (data points available/total possible data points)
1	Devices not sited	Devices not sited
2	100% (64/64)	11% (3/28)^a^
3	100% (112/112)	42% (31/74)^a^
4	100% (143/143)	69% (85/124)^a^
5	100% (156/156)	72% (112/156)^a^
6	100% (156 /156)	89% (138/156)^a^
7	100% (156/156)	100% (156/156)
8	100% (145/145)	99% (143/145)
9	100% (136/136)	99% (135/136)
10	100% (132/132)	99% (131/132)

^a^Adjusted Fisher exact test for success rate between devices (*P* < 0.005).

Variations in sensor on and off timings, as well as the sequential placement of sensors, caused differences in total possible data points from minute to minute and between devices, respectively.

### Oxygen saturation measurement

**Fig. 3:**
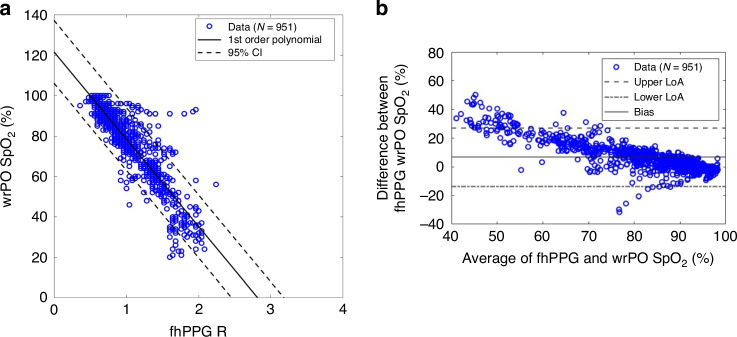
Cohort-level comparison of wrPO SpO_2_ versus fhPPG readings. **a** Correlation plot showing wrPO SpO_2_ against fhPPG R values. **b** Bland–Altman analysis comparing SpO_2_ values derived from fhPPG R (using Eq. 3) with wrPO SpO_2_.

A first-order polynomial regression fitted to the pooled data SpO_2_, from wrPO plotted against fhPPG R (Eq. 2), produced a regression line with a slope (intercept) of −43 (122) (Fig. 3a). The pooled correlation plots indicated an RMSE of 8% and Spearman’s correlation coefficient (*r*) of 0.88. Further, the BA analysis of the pooled data comparing fhPPG and wrPO SpO_2_ demonstrated a positive bias of 6.7% with limits of agreement at 20.4% (upper LoA = 27% and lower LoA *=* −13.7%) and is displayed in Fig. 3b.

Cohort-level SpO_2_ data from fhPPG and wrPO were compared (Fig. 4, Supplementary Table 3). At 2 min of life, the median (IQR) SpO_2_ for fhPPG was 82% (2%) and for wrPO was 77% (3%). The SpO_2_ levels gradually increased reaching over 90% at 10 min of life for both devices. There were significant differences found in SpO_2_ measured by fhPPG and wrPO between minutes 3 and 8 (*p* < 0.005). Infant-level time-varying HR and SpO_2_ plots, as well as correlation plots, are displayed in Supplementary Figs. 3–15.

**Fig. 4:**
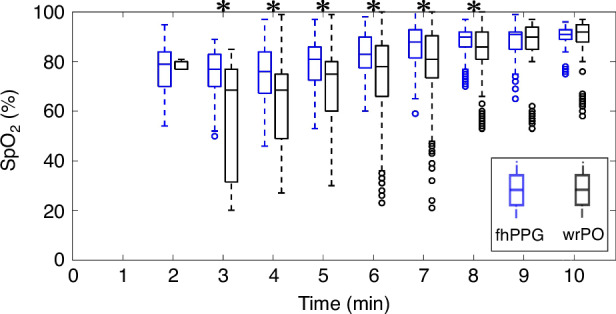
Cohort-level SpO_2_ plots recorded over 10 min post-birth. Box and whisker plots of cohort-level SpO_2_ recorded over 10 min post-birth as measured by fhPPG (blue) and wrPO (black) (devices are offset for clarity). *Adjusted Wilcoxon rank sum for success rate between devices (*P* < 0.005).

To ensure confidence and assess the validity of the generated fhPPG SpO_2_ readings, we compared HRs calculated from fhPPG against ECG to confirm the quality of the fhPPG signals. Example plots of raw signals from accepted and rejected PPG signal windows based on HR assessment are shown in Supplementary Figs. 16–21. Of note, we did not perform this validation for wrPO as we did not have access to the raw signals or signal quality index from the device.

## Discussion

The aim of this study was to establish the feasibility and compare HR and SpO_2_ data measured at two different locations (forehead vs wrist) in newborn infants immediately following birth. To our knowledge, this is the first published study to report SpO_2_ measurements from the newborn’s forehead within the first 10 min after birth using a PPG sensor. We have shown the feasibility of measuring both the HR and SpO_2_ using an fhPPG sensor on the forehead of healthy newborn infants immediately after ECS birth and that these measures correlate with peripheral measures.

In an earlier two-phase study, we assessed the accuracy and reliability of the fhPPG sensor for HR in NICU and delivery room settings where the fhPPG demonstrated a good correlation with paired ECG HR (*r* = 0.94).^21^ Similarly in the current study, HR data were measured using two PPG sensors, on the right wrist and forehead, against the gold standard chest ECG. From 2 min of age, there were no significant differences in HR between fhPPG and ECG throughout the recording. However, only 11% of possible wrPO HR measurements were reported at 2 min, all were <50 bpm despite the infants being well and not in need of support. Overall, fhPPG did not report an HR < 100 bpm throughout the recordings unlike ECG (*n* = 6) and wrPO (*n* = 13), although which device is correct isn’t possible to determine from this study. An HR < 100 bpm is important as these were well infants not requiring any form of support and if the attending team had used the reported HR it could have influenced their decision making to support these infants. The poor HR wrPO accuracy measures (lower PPA and higher RMSE), and potential episodes of <100 bpm, could reflect weaker peripheral perfusion at birth, as reported with similar wrPO vs ECG studies after birth.^25,26^ Low perfusion at the wrist during the newborn transition may have resulted in a reduced signal-to-noise ratio (due to decreased AC pulsatile amplitude relative to the constant background noise), leading to a difference in HR estimates between the wrist and forehead.

We observed that the SpO_2_ slowly rises, regardless of the measurement site, during the first few minutes of life until reaching a plateau >90%.^8,9^ A statistically significant difference in SpO_2_ was measured between the forehead and wrist, between 3 and 8 min, with the fhPPG probe producing higher SpO_2_ values than the wrPO. This finding is further supported by the systemic positive bias revealed in the BA analysis, indicating consistently higher SpO_2_ estimates from fhPPG than wrPO across measurements. The initial few moments after birth are a critical time for physiological adaptation with the blood supply to the brain, from the carotid arteries which also supply the forehead, taking precedence over other less important organs and peripheries which may have caused differences in SpO_2_ at these two measurement sites.^14,19^ In addition, we observed less variability with fhPPG than wrPO SpO_2_ throughout the recorded time and a strong correlation between fhPPG R and wrPO SpO_2_. Despite the theoretical physiological perfusion benefits of PPG forehead over peripheral monitoring during the transition, the present study does not allow us to identify which device is closer to the true arterial oxygen saturation.

Numerous studies have investigated the precision of SpO_2_ measurement in adults at the forehead and compared it to peripheral sites. Some of these studies, focusing on patients with conditions such as hypothermia,^27^ peripheral vasoconstriction,^14^ low cardiac index,^28^ vascular surgery,^29^ during emergency transport,^30^ or patients requiring vasopressor therapy,^31^ concluded that the forehead sensor provided better quality (i.e., lower variability) during SpO_2_ monitoring. However, other studies have reported that the forehead does not always outperform finger-based pulse oximetry.^32–34^ In our study, we observed strong agreement between fhPPG HR and ECG HR, characterised by high positive percent agreement, and lower root mean squared error, compared to wrPO HR. These findings support further exploration of the measurement of SpO_2_ using fhPPG as there appear to be benefits over conventional placed peripheral PO devices.

Several other factors may have contributed to this distinct difference in SpO_2_ at these two sites. The forehead region is covered by a thin layer of skin with a higher density of blood vessels and very little subcutaneous fat, unlike the peripheries of term babies, so the reflection of the optical signal from the forehead region is generally strong.^35^ Peripheral sites, such as fingers, toes or the wrist, exhibit a higher tendency for conditions such as vasoconstriction, low perfusion and motion artefact, which consequently lead to low amplitude AC pulsatile PPG signals.^13^ Another reason for the difference in the forehead and wrist SpO_2_ could be due to the difference in the way fhPPG, and wrPO estimated the SpO_2_. Understandably, the measurement approach is different from instrument to instrument, which may have caused a difference in readings. With regards to the forehead (fhPPG), SpO_2_ was computed by deploying the standard Texas Instruments equation (Eq. 3) once the *R*-value was determined. This equation is a linear approximation of the empirical calibration curve previously used in other studies, however, alternative non-linear models may also be more appropriate.^15,36^ Ideally, the SpO_2_ algorithm would be developed with comparison to paired arterial saturations taken from blood but this isn’t feasible in the immediate newborn period. Therefore, Eq. 3 was used as a starting point to determine SpO_2_, and while the higher SpO_2_ reported at the forehead is plausible, it should be treated with caution, as this equation may not represent the precise calibration curve for this sensor. Future research will focus on validating these findings, potentially through controlled desaturation studies in adults to compare SpO_2_ variability at the forehead and wrist.

### Limitations

Whilst this is the first study to try and measure forehead SpO_2_ in the newborn during the immediate post-birth transition period it does have some limitations. The algorithm deployed for estimating the fhPPG SpO_2_ was not formally developed with newborn arterial oxygen saturation measurements. The sample size was relatively small and included only healthy term newborns born by ECS so requires studying during high-risk births where infants commonly experience poor perfusion and hypoxia. The test protocol involved first placing the fhPPG sensor, followed by the ECG and wrPO sensor a few seconds after birth, resulting in a delay between sensor data. We cannot exclude bias with time to signal acquisition because of sensor placement although there are clear differences for more than five minutes for SpO_2_ measures.

## Conclusion

The forehead PPG sensor demonstrates good HR agreement with the ECG HR. The SpO_2_ measures indicate higher values at the forehead compared to a peripherally sited PO. The study supports better perfusion of the forehead region over the peripheral region as demonstrated by more accurate HR measures and less SpO_2_ variability. The significant differences in SpO_2_ measures on the forehead and wrist need further exploration to understand which better reflects true arterial oxygen saturation. The use of a PPG sensor on the newborn forehead in the delivery room could provide a novel alternative for measuring not only HR but also SpO_2_ at birth and warrants further investigation.

## Supplementary information

Supplemental Document_revised

## Data Availability

The datasets presented in this article are not readily available for public access as they will be further used for commercial purposes by Surepulse Medical Ltd. The data will be made publicly available at a future date, please contact the authors. Requests to access the datasets should be directed to Barrie Hayes-Gill, barrie.hayes-gill@nottingham.ac.uk.
