# Supplementary material for: Comparing peripheral limb and forehead vital sign monitoring in newborn infants at birth
Source: Pediatr Res. 2024 Oct 17;99(2):598–603. doi: 10.1038/s41390-024-03651-0 (PMC12956579; doi:10.1038/s41390-024-03651-0)
Supplement: Supplementary file 1 — Supplemental Document_revised [file 41390_2024_3651_MOESM1_ESM.pdf]

Supplemental Tables and Figures

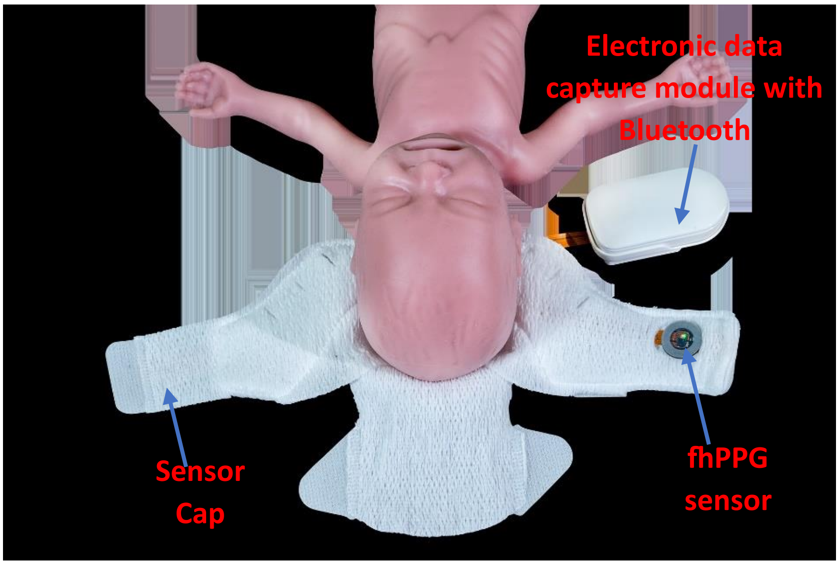

Figure 1 A photograph of the fhPPG sensor sited on a mannequin, illustrating its placement on a newborn.

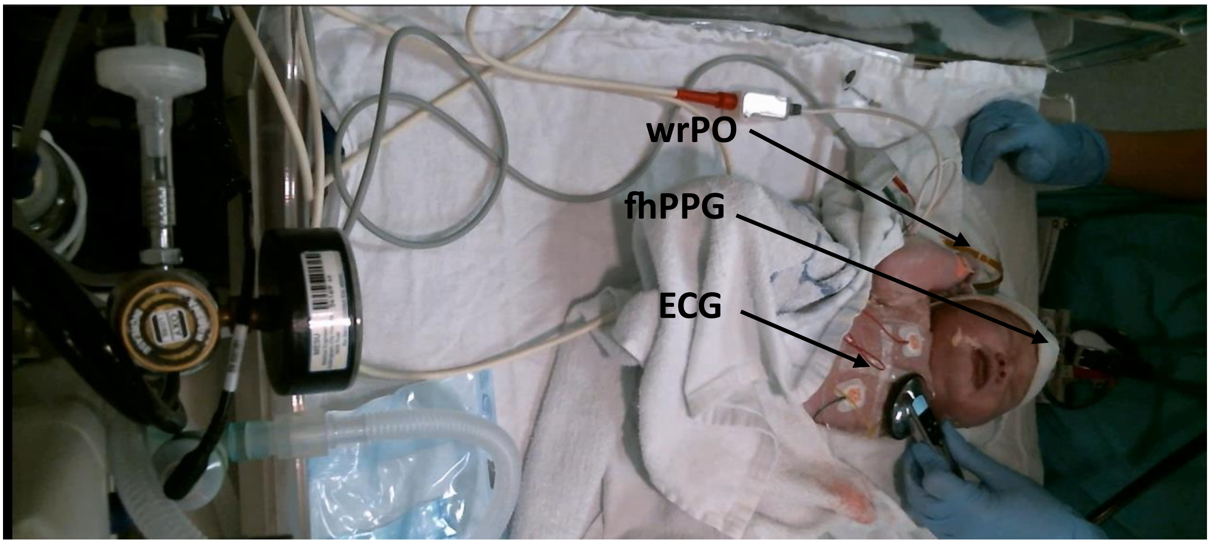

Figure 2 A snapshot of the entire set up that shows the arrangement of all the equipment's in place and the baby on the resuscitaire.

Table 1 Placement times (in seconds) relative to the birth time for all three sensors ECG, fhPPG and wrPO sensors. The variability was dependent on how long the obstetric team spent time showing the newborn to the mother.

Infant	ECG	fhPPG	wrPO
02	95	66	77
05	73	59	92
06	142	136	172
07	149	139	172
09	215	201	234
10	78	65	98
11	89	79	102
15	157	139	175
16	219	204	232
17	78	69	99
18	213	201	234
19	149	129	159
20	112	128	149

Table 2 Median (IQR) HR (bpm) from ECG, fhPPG and wrPO at each minute. Comparison of ECG HR for cohort - level differences for fhPPG and wrPO.

Time (min)	ECG HR Median (IQR)	fhPPG HR Median (IQR)	fhPPG p value	wrPO HR Median (QIR)	wrPO p value
1	-	-	-	-	-
2	166(36)	155 (28)	0.64	36 (3)	<0.01
3	149 (26.2)	158 (24)	0.70	167 (43)	0.19
4	151 (23.7)	150 (20.5)	0.86	143 (28)	0.07
5	149 (22)	148 (21)	0.21	147 (25.5)	0.14
6	144 (13)	145 (14)	0.50	143 (17)	0.64
7	143 (13.5)	143 (15)	0.61	143 (14)	0.57
8	141.5 (12)	143 (10)	0.41	141 (13)	0.62
9	142 (12)	141 (10)	0.69	142 (14)	0.37
10	142 (11)	143 (11)	0.12	142 (12.7)	0.88

Table 3 presents a minute-by-minute cohort-level p-values for SpO_2, along with a comparison of the median (IQR) SpO_2 from wrPO, and fhPPG.

Time (min)	Median fhPPG SpO_2 (%)	Median wrPO SpO_2 (%)	p value for SpO_2
1	-	-	-
2	82 (2)	77 (3)	0.2
3	82 (22)	70 (46)	<0.01
4	77 (18)	68 (28)	<0.01
5	84 (15)	75 (20)	<0.01
6	84 (12)	78 (21)	<0.01
7	89 (12)	82 (18)	<0.01
8	90 (6)	86 (11)	<0.01
9	91 (7)	90 (9)	0.52
10	91 (5)	92 (7)	0.03

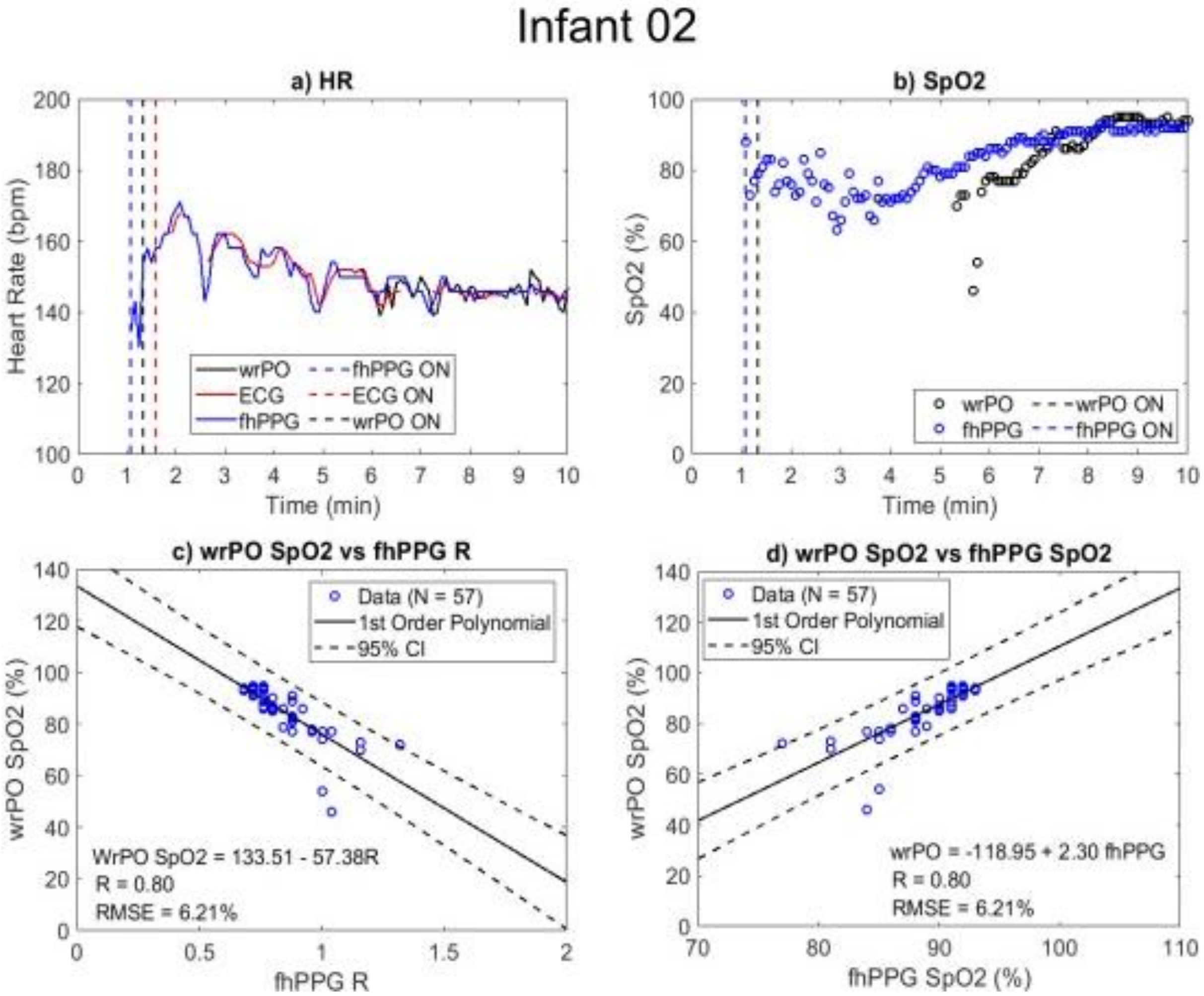

Figure 3 Shows signals recorded during the first 10 minutes post-birth for infant 02: (a) HR and (b) SpO_2. Additionally, (c) presents the correlation plot of wrPO SpO_2 against fhPPG R, while (d) shows the correlation plot of wrPO SpO_2 against fhPPG SpO_2.

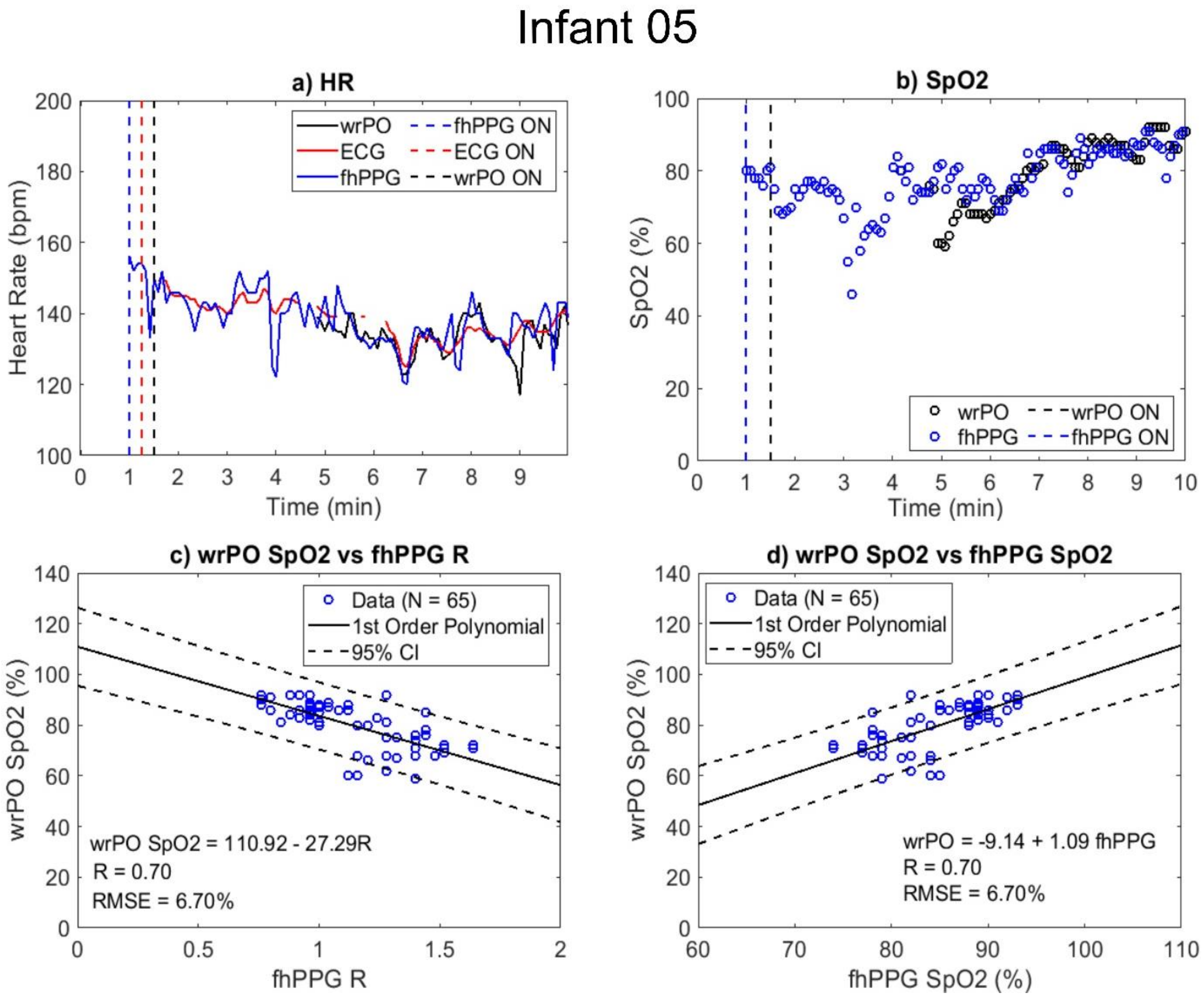

Figure 4 Shows signals recorded during the first 10 minutes post-birth for infant 05: (a) HR and (b) SpO_2. Additionally, (c) presents the correlation plot of wrPO SpO_2 against fhPPG R, while (d) shows the correlation plot of wrPO SpO_2 against fhPPG SpO_2.

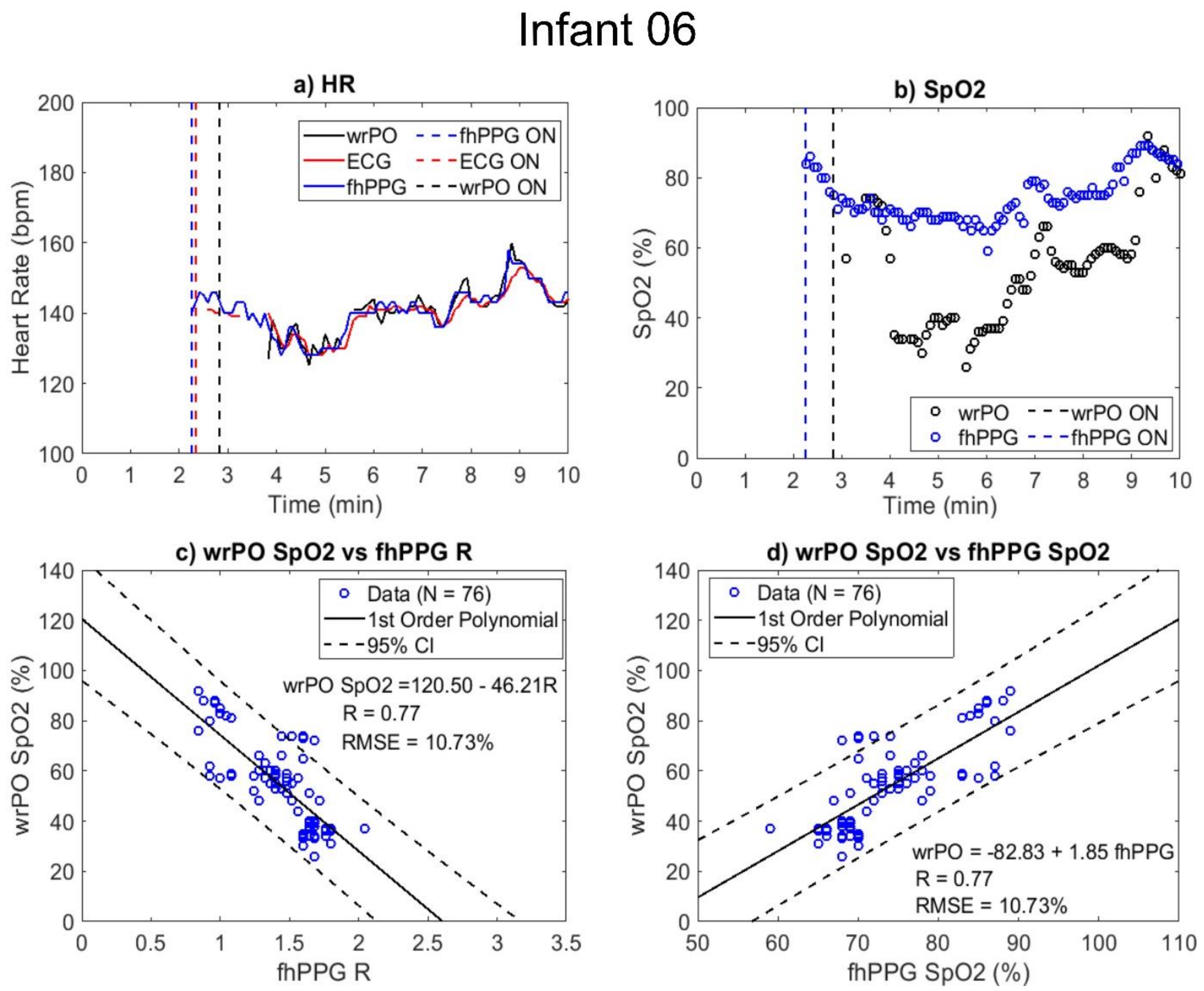

Figure 5 Shows signals recorded during the first 10 minutes post-birth for infant 06: (a) HR and (b) SpO_2. Additionally, (c) presents the correlation plot of wrPO SpO_2 against fhPPG R, while (d) shows the correlation plot of wrPO SpO_2 against fhPPG SpO_2.

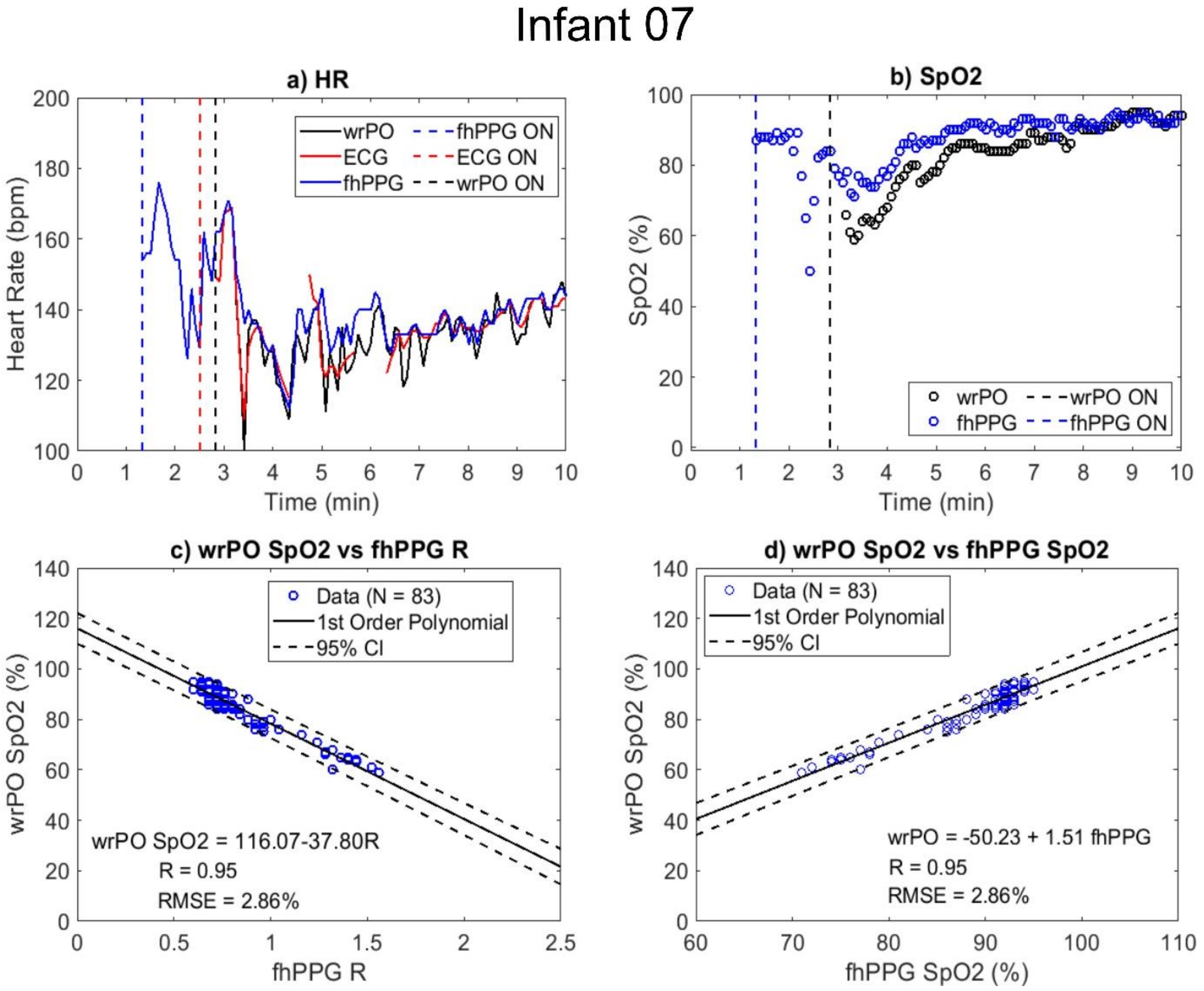

Figure 6 Shows signals recorded during the first 10 minutes post-birth for infant 07: (a) HR and (b) SpO_2. Additionally, (c) presents the correlation plot of wrPO SpO_2 against fhPPG R, while (d) shows the correlation plot of wrPO SpO_2 against fhPPG SpO_2.

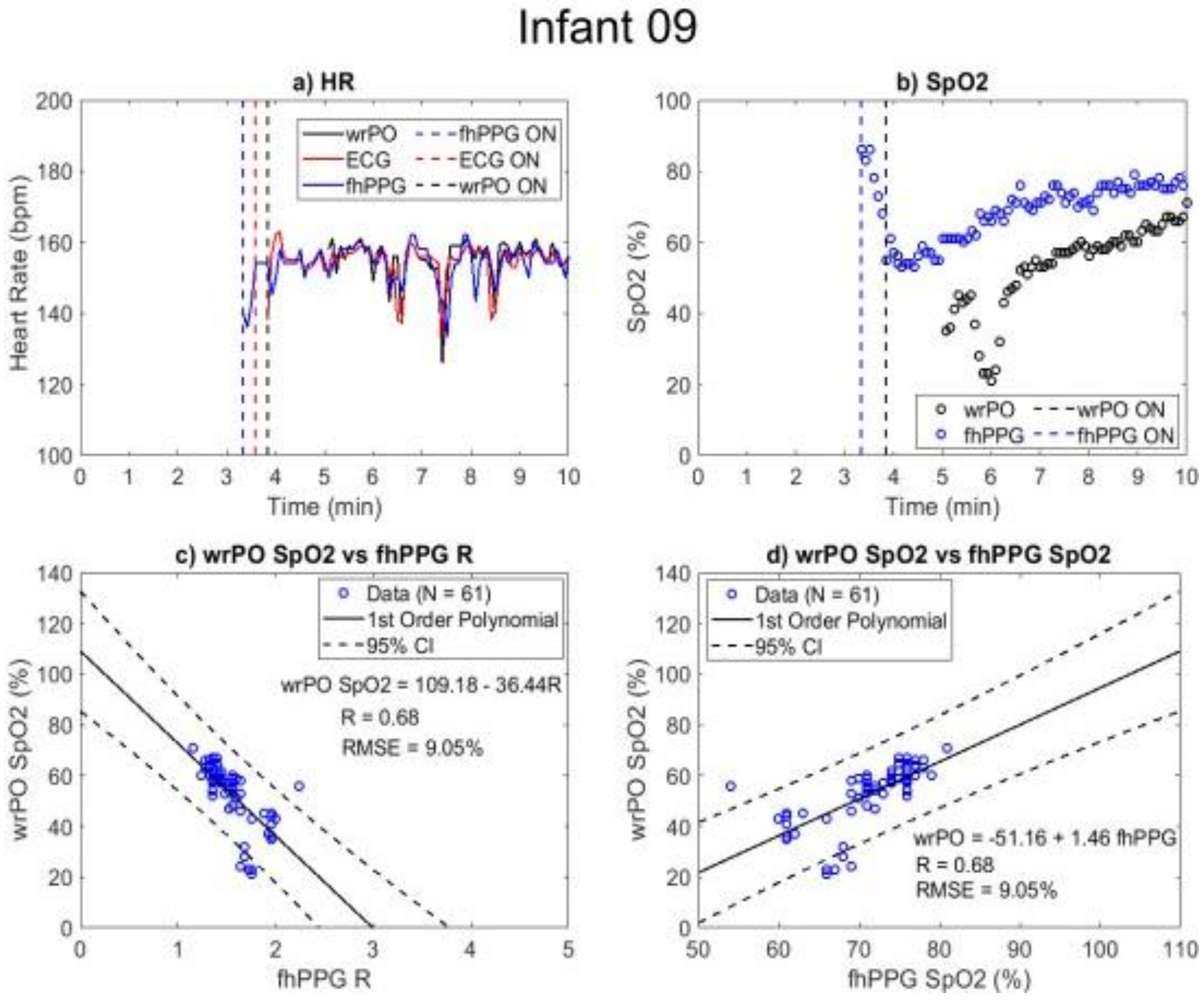

Figure 7 Shows signals recorded during the first 10 minutes post-birth for infant 09: (a) HR and (b) SpO_2. Additionally, (c) presents the correlation plot of wrPO SpO_2 against fhPPG R, while (d) shows the correlation plot of wrPO SpO_2 against fhPPG SpO_2.

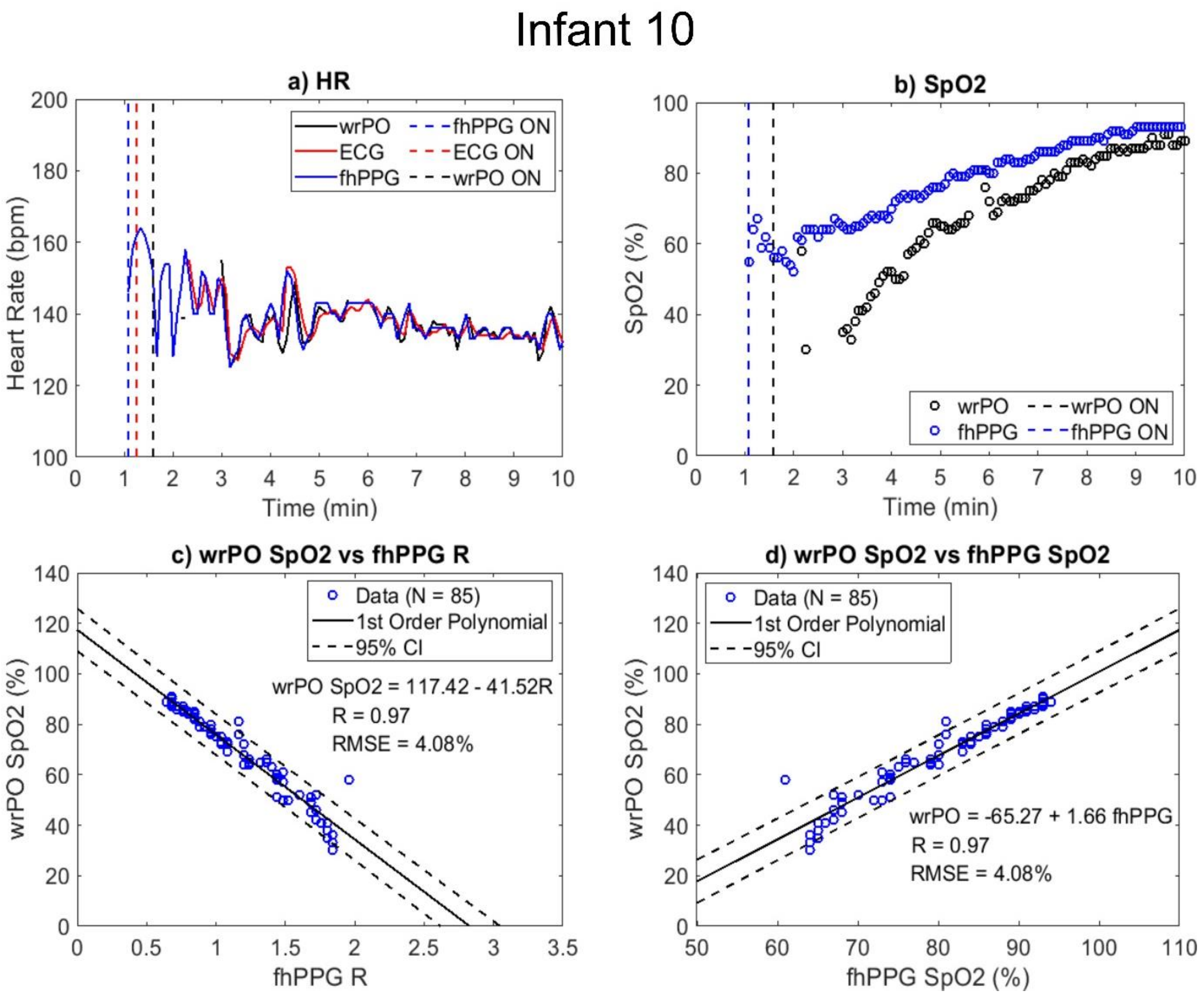

Figure 8 Shows signals recorded during the first 10 minutes post-birth for infant 10: (a) HR and (b) SpO_2. Additionally, (c) presents the correlation plot of wrPO SpO_2 against fhPPG R, while (d) shows the correlation plot of wrPO SpO_2 against fhPPG SpO_2.

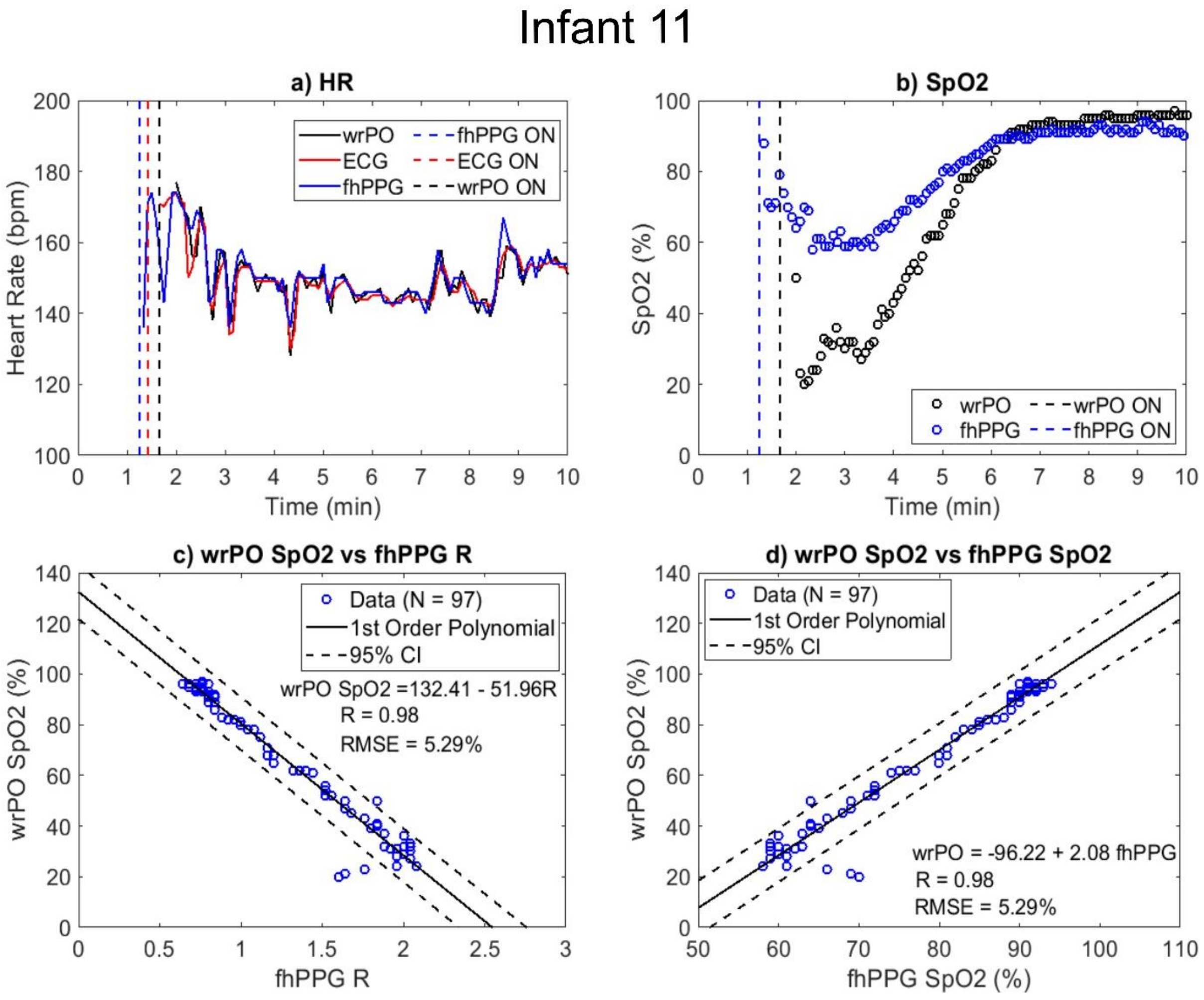

Figure 9 Shows signals recorded during the first 10 minutes post-birth for infant 11: (a) HR and (b) SpO_2. Additionally, (c) presents the correlation plot of wrPO SpO_2 against fhPPG R, while (d) shows the correlation plot of wrPO SpO_2 against fhPPG SpO_2.

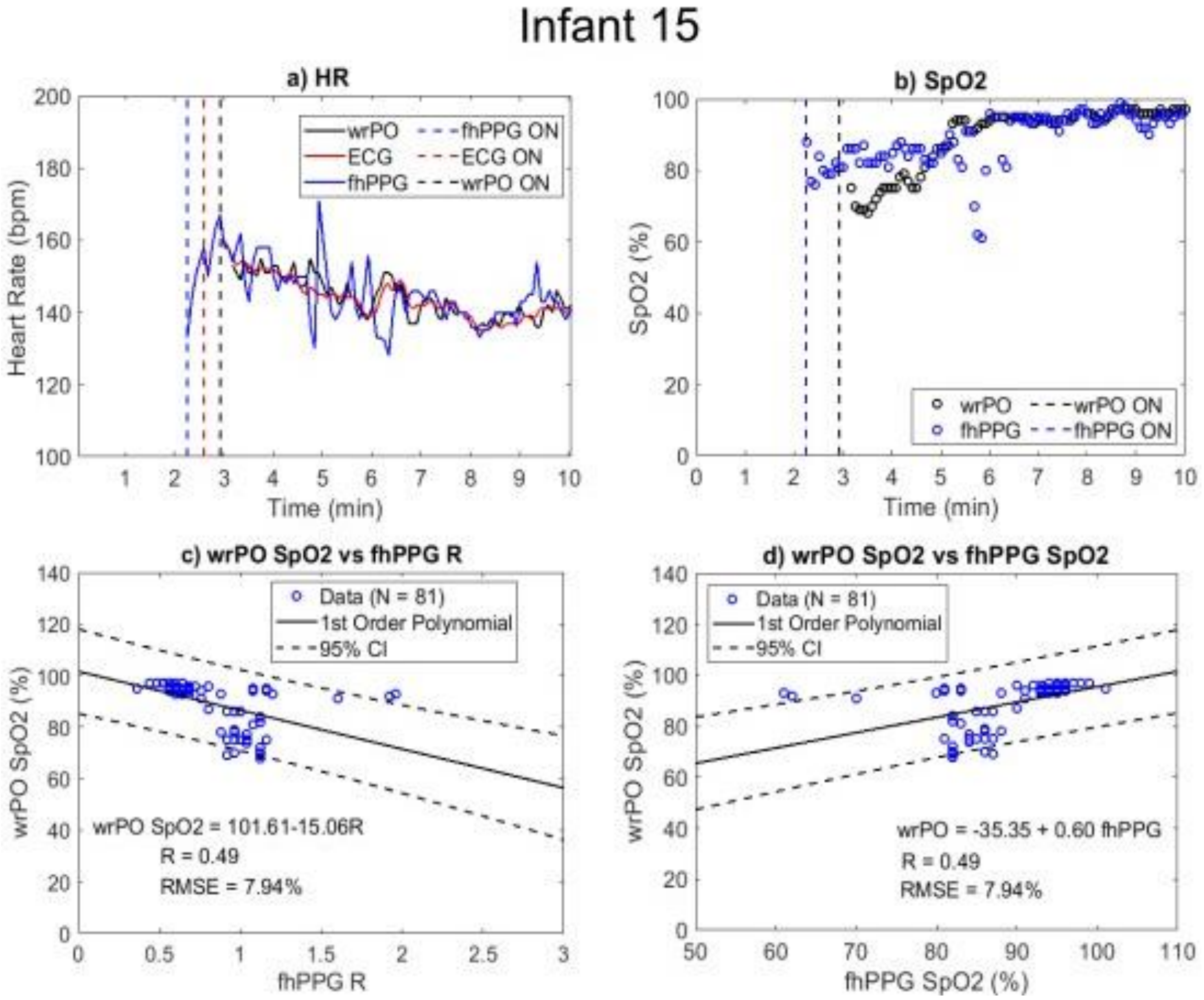

Figure 10 Shows signals recorded during the first 10 minutes post-birth for infant 15: (a) HR and (b) SpO_2. Additionally, (c) presents the correlation plot of wrPO SpO_2 against fhPPG R, while (d) shows the correlation plot of wrPO SpO_2 against fhPPG SpO_2.

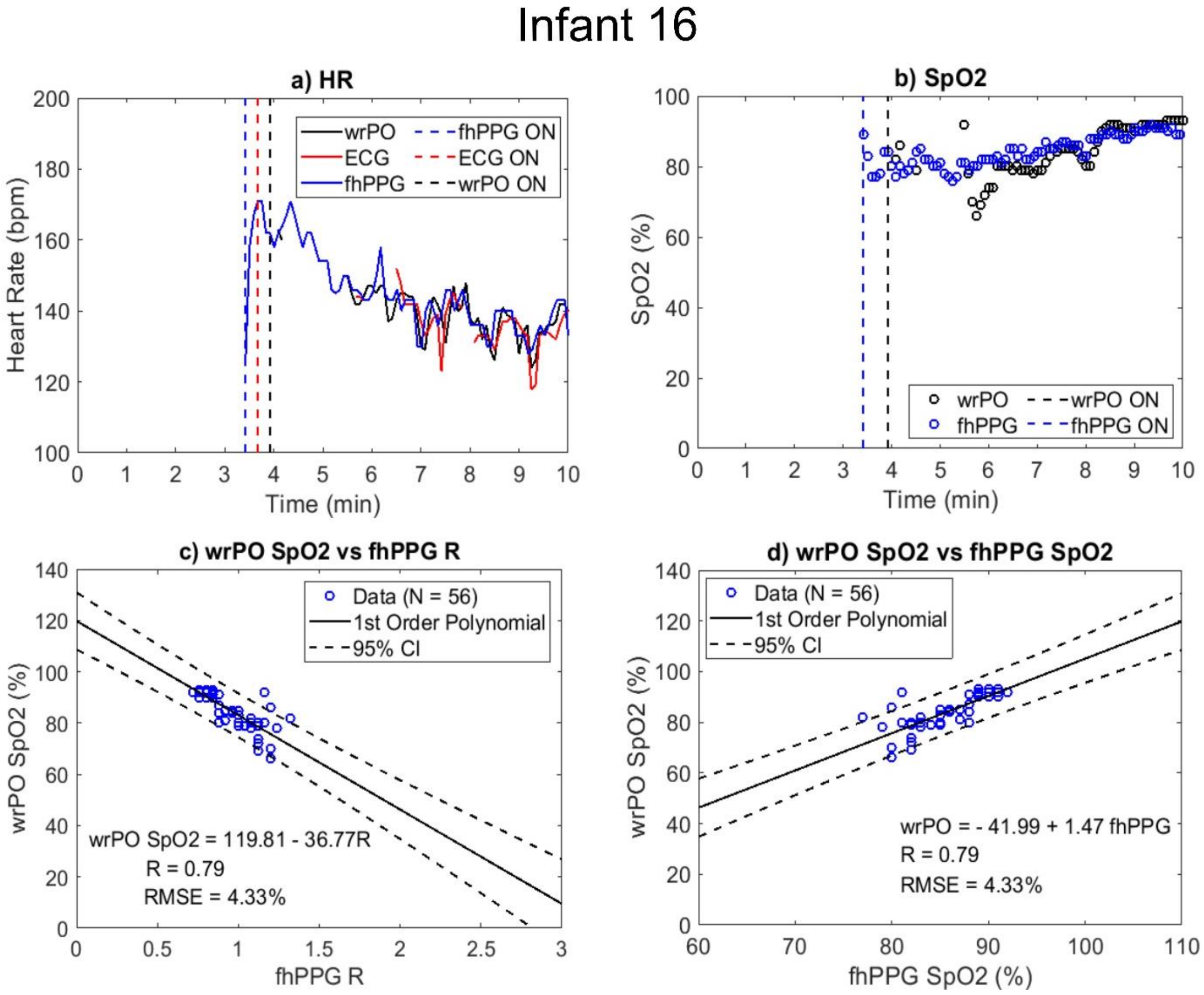

Figure 11 Shows signals recorded during the first 10 minutes post-birth for infant 16: (a) HR and (b) SpO_2. Additionally, (c) presents the correlation plot of wrPO SpO_2 against fhPPG R, while (d) shows the correlation plot of wrPO SpO_2 against fhPPG SpO_2.

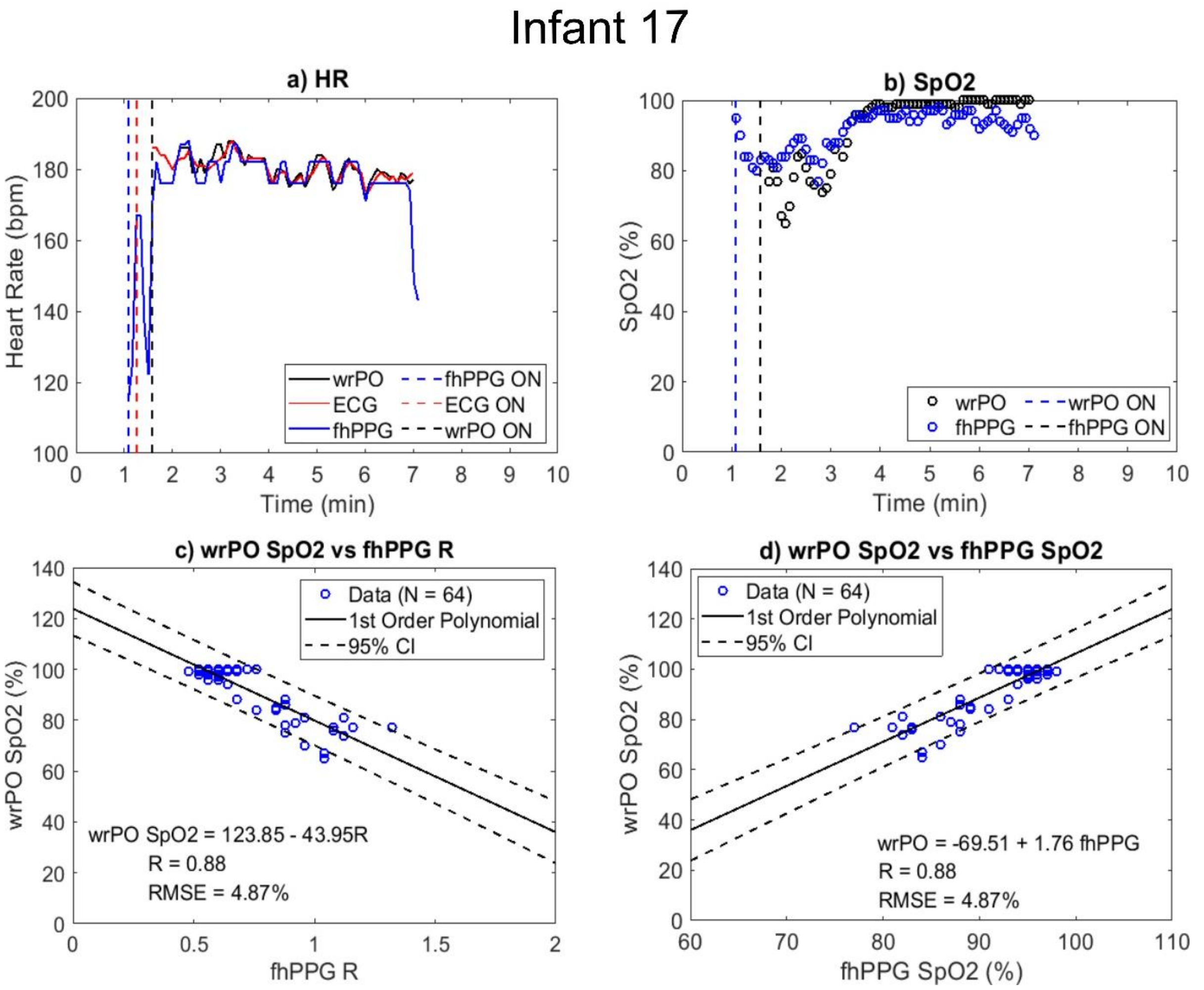

Figure 12 Shows signals recorded during the first 10 minutes post-birth for infant 17: (a) HR and (b) SpO_2. Additionally, (c) presents the correlation plot of wrPO SpO_2 against fhPPG R, while (d) shows the correlation plot of wrPO SpO_2 against fhPPG SpO_2.

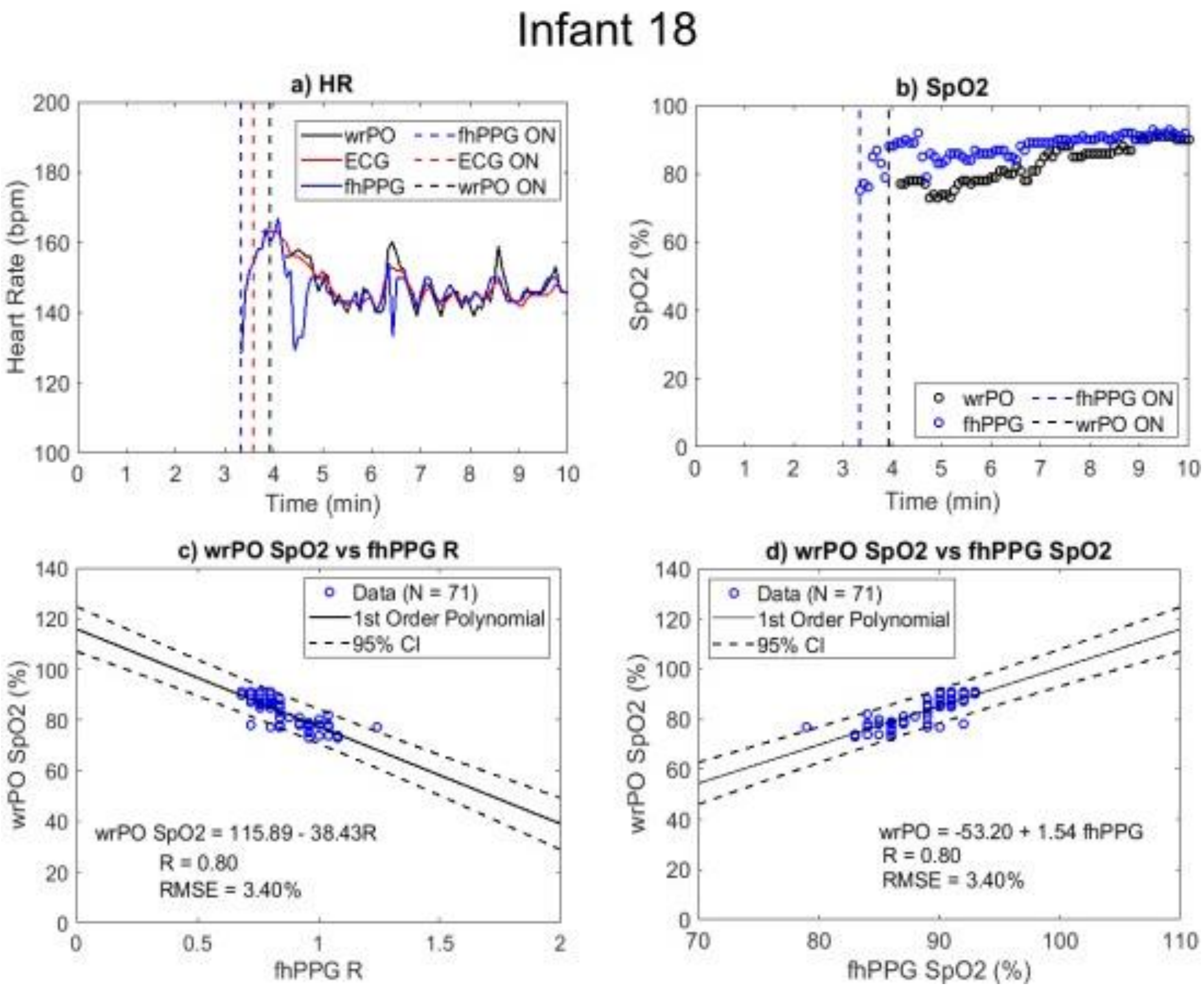

Figure 13 Shows signals recorded during the first 10 minutes post-birth for infant 18: (a) HR and (b) SpO_2. Additionally, (c) presents the correlation plot of wrPO SpO_2 against fhPPG R, while (d) shows the correlation plot of wrPO SpO_2 against fhPPG SpO_2.

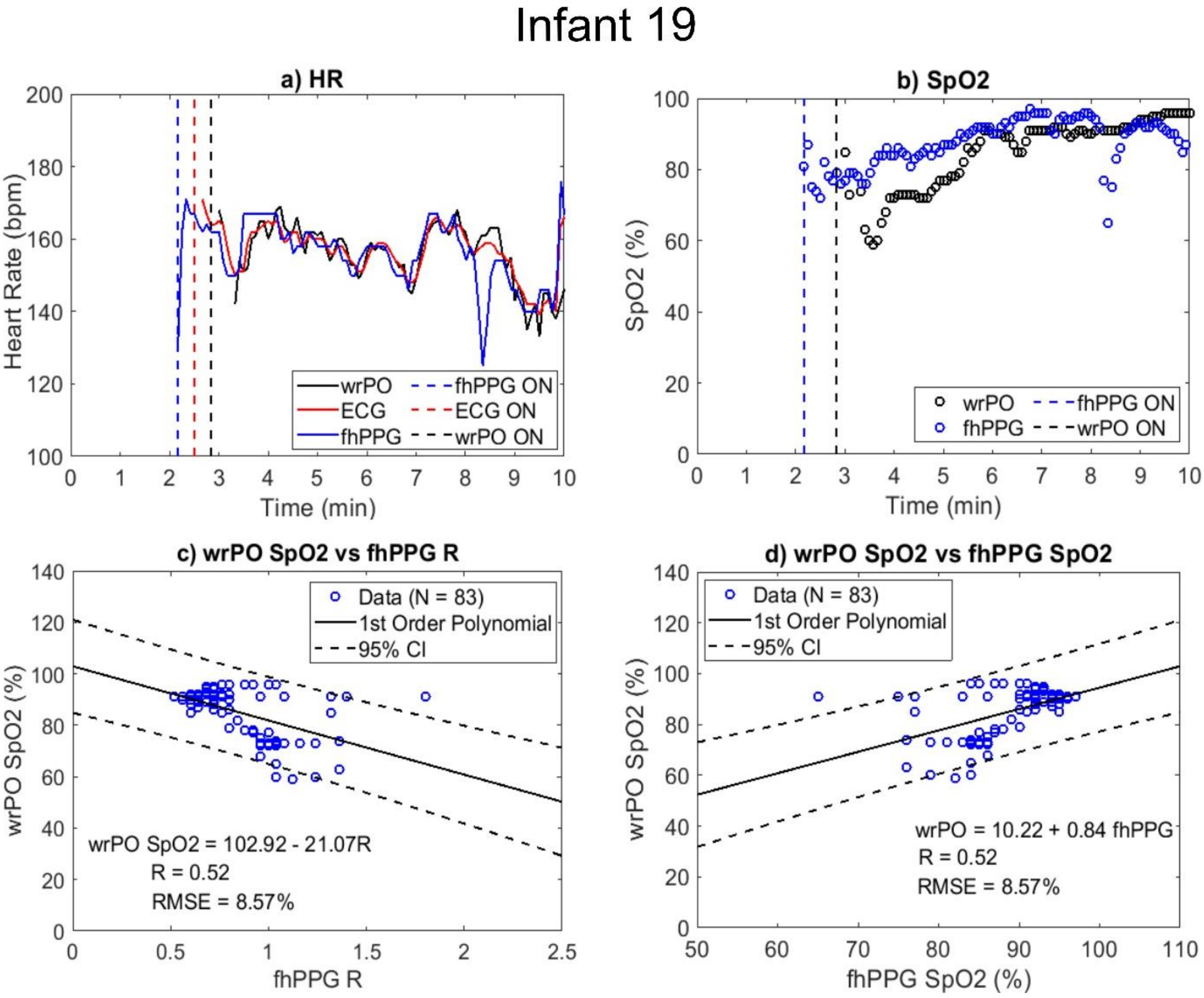

Figure 14 Shows signals recorded during the first 10 minutes post-birth for infant 19: (a) HR and (b) SpO_2. Additionally, (c) presents the correlation plot of wrPO SpO_2 against fhPPG R, while (d) shows the correlation plot of wrPO SpO_2 against fhPPG SpO_2.

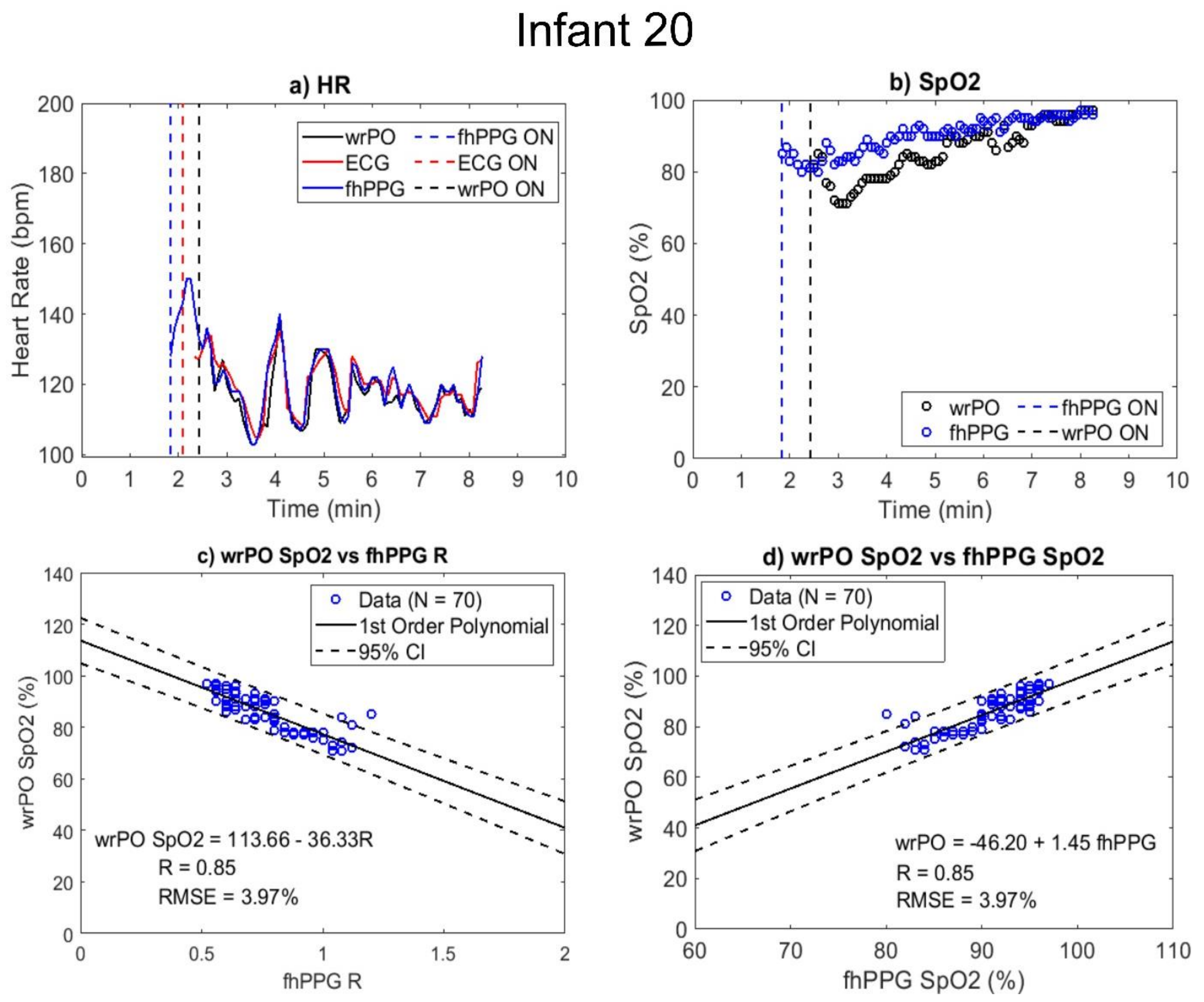

Figure 15 Shows signals recorded during the first 10 minutes post-birth for infant 20: (a) HR and (b) SpO_2. Additionally, (c) presents the correlation plot of wrPO SpO_2 against fhPPG R, while (d) shows the correlation plot of wrPO SpO_2 against fhPPG SpO_2.

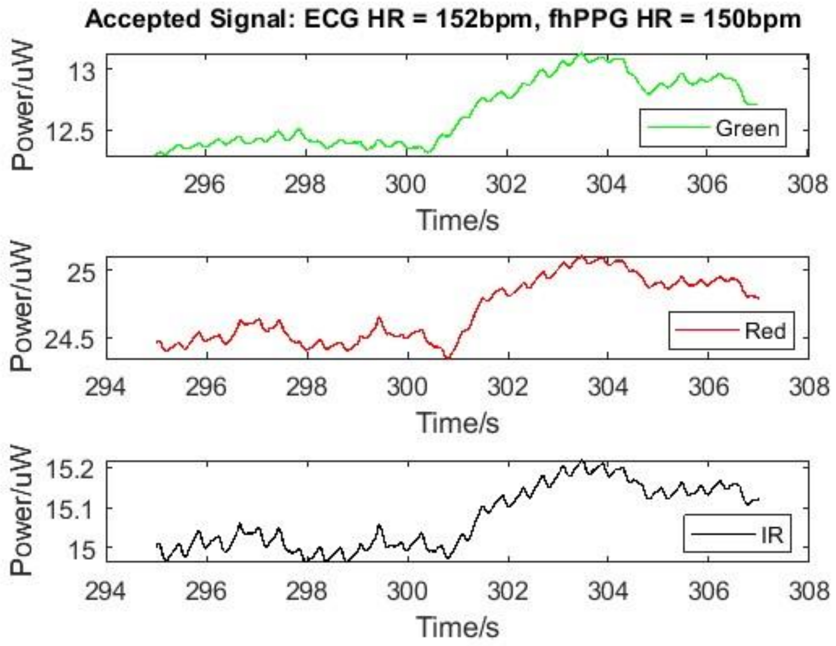

Figure 16 Shows real-time plot example #1 of an accepted signal window based on the HR assessment.

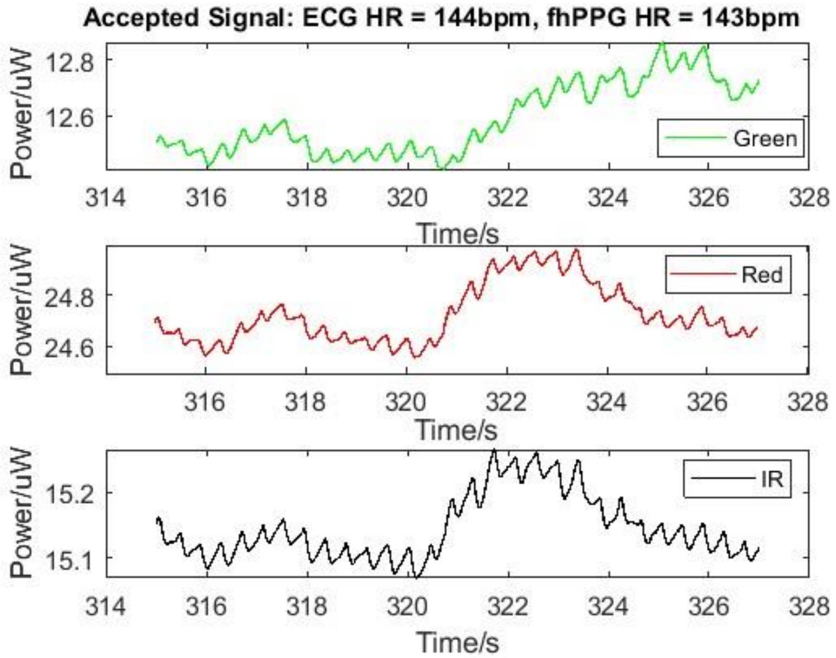

Figure 17 Shows real-time plot example #2 of an accepted signal window based on the HR assessment.

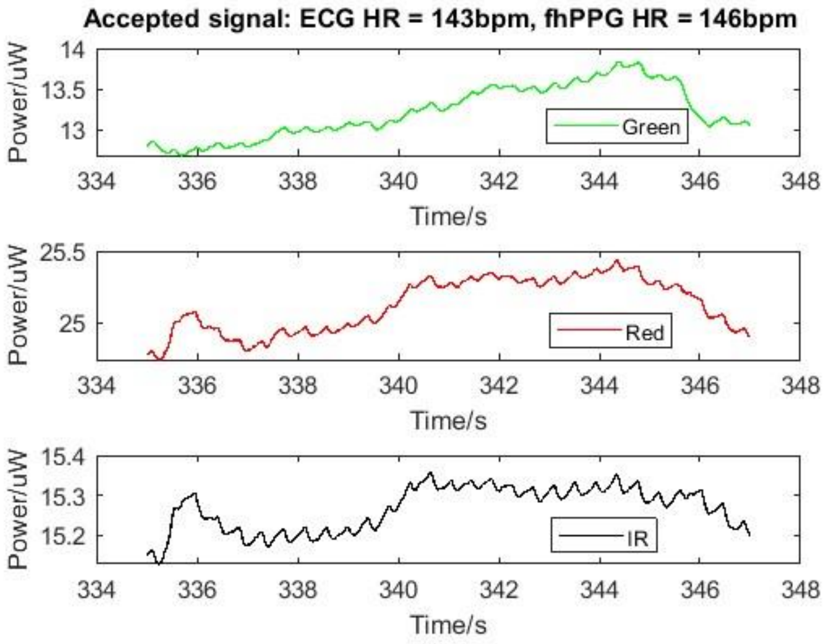

Figure 18 Shows real-time plot example #3 of an accepted signal window based on the HR assessment.

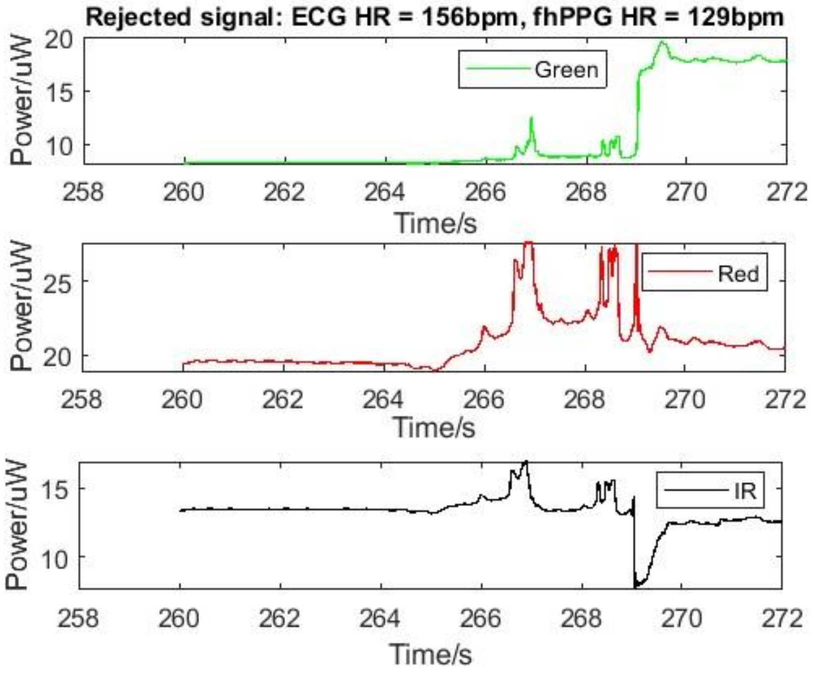

Figure 19 Shows real-time plot example #1 of a rejected signal window based on the HR assessment.

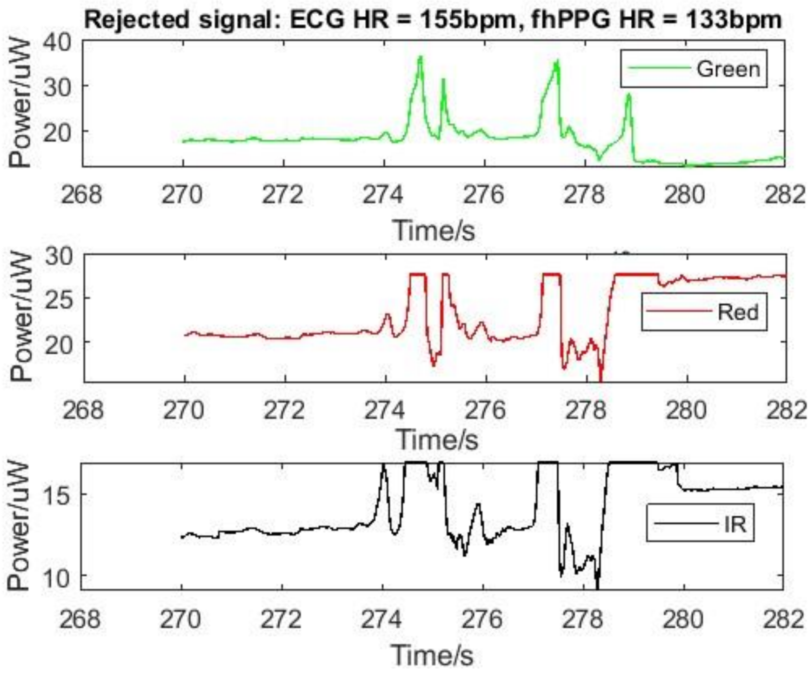

Figure 20 Shows real-time plot example #2 of a rejected signal window based on the HR assessment.

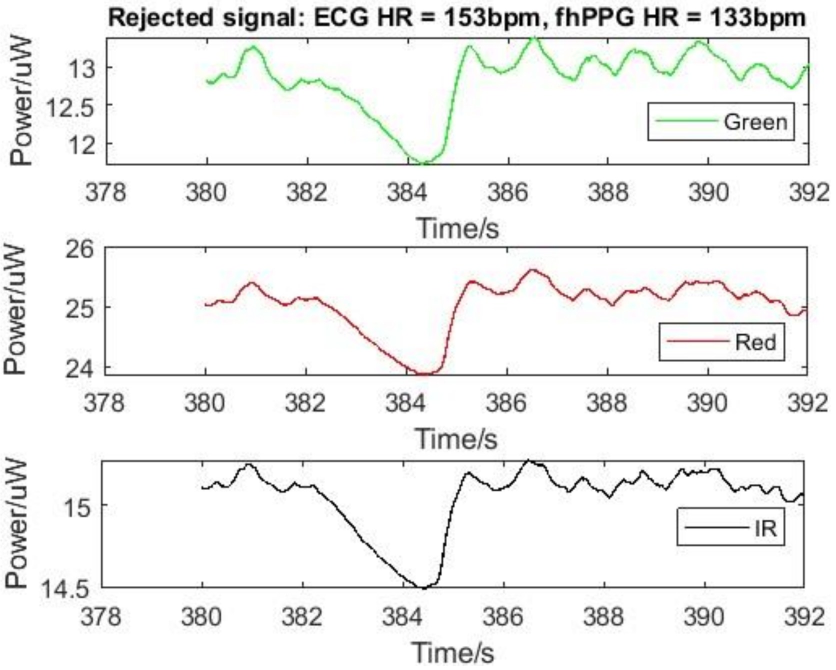

Figure 21 Shows real-time plot example #3 of a rejected signal window based on the HR assessment.
